# 2-(4-Bromo­phen­yl)-*N*-(3-chloro-4-fluoro­phen­yl)acetamide

**DOI:** 10.1107/S1600536812032977

**Published:** 2012-07-25

**Authors:** Hoong-Kun Fun, Ching Kheng Quah, Prakash S. Nayak, B. Narayana, B. K. Sarojini

**Affiliations:** a X-ray Crystallography Unit, School of Physics, Universiti Sains Malaysia, 11800 USM, Penang, Malaysia; b Department of Studies in Chemistry, Mangalore University, Mangalagangotri 574 199, India; c Department of Chemistry, P. A. College of Engineering, Nadupadavu, Mangalore 574 153, India

## Abstract

In the title compound, C_14_H_10_BrClFNO, the benzene rings form a dihedral angle of 64.0 (2)°. In the crystal, mol­ecules are linked *via* inter­molecular N—H⋯O, C—H⋯O, C—H⋯Cl and C—H⋯F hydrogen bonds into layers parallel to (001). The crystal was refined as a merohedral twin with a 0.935 (114):0.065 (14) domain ratio.

## Related literature

For general background to the title compound and for related structures, see: Fun *et al.* (2011*a* ▶,*b* ▶, 2012*a* ▶,*b* ▶). For the stability of the temperature controller used in the data collection, see: Cosier & Glazer (1986 ▶).
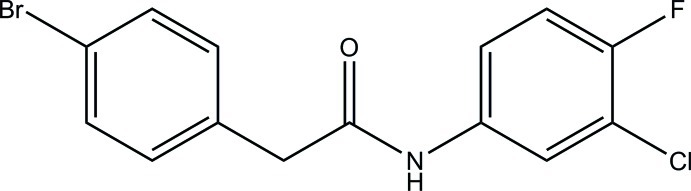


## Experimental

### 

#### Crystal data

C_14_H_10_BrClFNO
*M* *_r_* = 342.59
Orthorhombic, 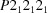

*a* = 4.9120 (5) Å
*b* = 6.3131 (6) Å
*c* = 42.517 (4) Å
*V* = 1318.4 (2) Å^3^
*Z* = 4
Mo *K*α radiation
μ = 3.32 mm^−1^
*T* = 100 K
0.30 × 0.17 × 0.07 mm

#### Data collection

Bruker SMART APEXII DUO CCD area-detector diffractometer
Absorption correction: multi-scan (*SADABS*; Bruker, 2009 ▶) *T*_min_ = 0.440, *T*_max_ = 0.806
10866 measured reflections
4737 independent reflections
4358 reflections with *I* > 2σ(*I*)
*R*_int_ = 0.032

#### Refinement

*R*[*F* ^2^ > 2σ(*F* ^2^)] = 0.054
*wR*(*F* ^2^) = 0.120
*S* = 1.15
4737 reflections
173 parameters
H-atom parameters constrained
Δρ_max_ = 0.86 e Å^−3^
Δρ_min_ = −1.82 e Å^−3^
Absolute structure: Flack (1983 ▶), 1929 Friedel pairs
Flack parameter: 0.065 (14)

### 

Data collection: *APEX2* (Bruker, 2009 ▶); cell refinement: *SAINT* (Bruker, 2009 ▶); data reduction: *SAINT*; program(s) used to solve structure: *SHELXTL* (Sheldrick, 2008 ▶); program(s) used to refine structure: *SHELXTL*; molecular graphics: *SHELXTL*; software used to prepare material for publication: *SHELXTL* and *PLATON* (Spek, 2009 ▶).

## Supplementary Material

Crystal structure: contains datablock(s) global, I. DOI: 10.1107/S1600536812032977/rz2791sup1.cif

Structure factors: contains datablock(s) I. DOI: 10.1107/S1600536812032977/rz2791Isup2.hkl

Supplementary material file. DOI: 10.1107/S1600536812032977/rz2791Isup3.cml

Additional supplementary materials:  crystallographic information; 3D view; checkCIF report

## Figures and Tables

**Table 1:** Hydrogen-bond geometry (Å, °)

*D*—H⋯*A*	*D*—H	H⋯*A*	*D*⋯*A*	*D*—H⋯*A*
N1—H1*N*1⋯O1^i^	0.88	1.97	2.844 (5)	172
C2—H2*A*⋯O1^ii^	0.95	2.58	3.321 (5)	135
C10—H10*A*⋯Cl1^iii^	0.95	2.67	3.583 (5)	160
C11—H11*A*⋯F1^iv^	0.95	2.52	3.443 (6)	165

Symmetry codes: (i) 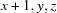; (ii) 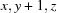; (iii) 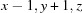; (iv) 
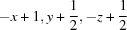
.

**Fig. 1.**
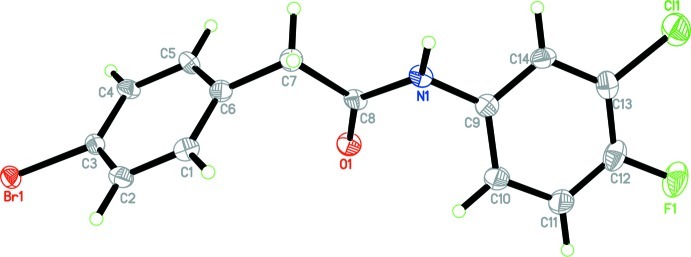
The molecular structure of the title compound showing 50% probability displacement ellipsoids for non-H atoms.

**Fig. 2.**
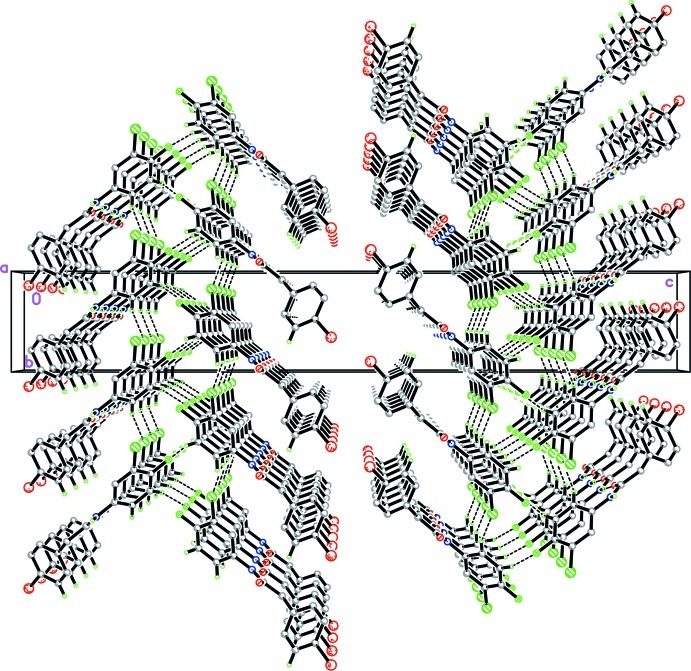
The crystal structure of the title compound, viewed along the a axis. H atoms not involved in hydrogen bonds (dashed lines) have been omitted for clarity.

## supplementary crystallographic information

### Comment

In continuation of our work on the synthesis of amides (Fun *et al.*, 2011*a*, 2011*b*, 2012*a*, 2012*b*), we report herein the crystal structure of the title compound.

In the title molecule (Fig. 1), the two benzene rings (C1-C6, C9-C14) form a dihedral angle of 64.0 (2)°. Bond lengths and angles are within normal ranges and are comparable to those found in related structures (Fun *et al.*, 2011*a*, 2011*b*, 2012*a*, 2012*b*). In the crystal structure (Fig. 2), molecules are linked *via* intermolecular N1–H1N1···O1, C2–H2A···O1, C10–H10A···Cl1 and C11–H11A···F1 hydrogen bonds (Table 1) into two-dimensional layers parallel to (001).

### Experimental

4-Bromophenylacetic acid (0.213 g, 1 mmol), 3-chloro-4-fluoroaniline (0.145 g, 1 mmol) and 1-ethyl-3-(3-dimethylaminopropyl)-carbodiimide hydrochloride (1.0 g, 0.01 mol) were dissolved in dichloromethane (20 mL). The mixture was stirred in presence of triethylamine at 273 K for about 3 h, poured into 100 mL of ice-cold aqueous hydrochloric acid with stirring and was then extracted thrice with dichloromethane. The organic layer was washed with a saturated NaHCO_3_ solution and brine solution, dried and concentrated under reduced pressure to give the title compound. Single crystals were grown from acetone and toluene (1:1 *v*/*v*) mixture by the slow evaporation method (m.p.: 415-417 K).

### Refinement

Atom H1N1 was located in a difference Fourier map and refined using a riding model with *U*_iso_(H) = 1.2 *U*_eq_(N) [N–H = 0.8825 Å]. The remaining H atoms were positioned geometrically and refined using a riding model with C–H = 0.95 or 0.99 Å and *U*_iso_(H) = 1.2 *U*_eq_(C). The crystal was refined as an inversion twin with a final refined BASF ratio of 0.935 (114):0.065 (14) for 1929 Friedel pairs.

### Crystal data

**Table:**

C_14_H_10_BrClFNO	*F*(000) = 680
*M**_r_* = 342.59	*D*_x_ = 1.726 Mg m^−^^3^
Orthorhombic, *P*2_1_2_1_2_1_	Mo *K*α radiation, λ = 0.71073 Å
Hall symbol: P 2ac 2ab	Cell parameters from 4232 reflections
*a* = 4.9120 (5) Å	θ = 3.5–31.8°
*b* = 6.3131 (6) Å	µ = 3.32 mm^−^^1^
*c* = 42.517 (4) Å	*T* = 100 K
*V* = 1318.4 (2) Å^3^	Plate, colourless
*Z* = 4	0.30 × 0.17 × 0.07 mm

### Data collection

**Table:**

Bruker SMART APEXII DUO CCD area-detector diffractometer	4737 independent reflections
Radiation source: fine-focus sealed tube	4358 reflections with *I* > 2σ(*I*)
Graphite monochromator	*R*_int_ = 0.032
φ and ω scans	θ_max_ = 32.5°, θ_min_ = 3.3°
Absorption correction: multi-scan (*SADABS*; Bruker, 2009)	*h* = −7→7
*T*_min_ = 0.440, *T*_max_ = 0.806	*k* = −9→9
10866 measured reflections	*l* = −46→63

### Refinement

**Table:**

Refinement on *F*^2^	Secondary atom site location: difference Fourier map
Least-squares matrix: full	Hydrogen site location: inferred from neighbouring sites
*R*[*F*^2^ > 2σ(*F*^2^)] = 0.054	H-atom parameters constrained
*wR*(*F*^2^) = 0.120	*w* = 1/[σ^2^(*F*_o_^2^) + (0.0038*P*)^2^ + 3.9457*P*] where *P* = (*F*_o_^2^ + 2*F*_c_^2^)/3
*S* = 1.15	(Δ/σ)_max_ = 0.001
4737 reflections	Δρ_max_ = 0.86 e Å^−^^3^
173 parameters	Δρ_min_ = −1.82 e Å^−^^3^
0 restraints	Absolute structure: Flack (1983), 1929 Friedel pairs
Primary atom site location: structure-invariant direct methods	Flack parameter: 0.065 (14)

### Special details

**Table:**

Experimental. The crystal was placed in the cold stream of an Oxford Cryosystems Cobra open-flow nitrogen cryostat (Cosier & Glazer, 1986) operating at 100.0 (1) K.
Geometry. All esds (except the esd in the dihedral angle between two l.s. planes) are estimated using the full covariance matrix. The cell esds are taken into account individually in the estimation of esds in distances, angles and torsion angles; correlations between esds in cell parameters are only used when they are defined by crystal symmetry. An approximate (isotropic) treatment of cell esds is used for estimating esds involving l.s. planes.
Refinement. Refinement of F^2^ against ALL reflections. The weighted R-factor wR and goodness of fit S are based on F^2^, conventional R-factors R are based on F, with F set to zero for negative F^2^. The threshold expression of F^2^ > 2sigma(F^2^) is used only for calculating R-factors(gt) etc. and is not relevant to the choice of reflections for refinement. R-factors based on F^2^ are statistically about twice as large as those based on F, and R- factors based on ALL data will be even larger.

### Fractional atomic coordinates and isotropic or equivalent isotropic displacement parameters (Å^2^)

**Table:**

	*x*	*y*	*z*	*U*_iso_*/*U*_eq_	
Br1	0.05032 (9)	1.14272 (6)	0.026647 (9)	0.01959 (9)	
Cl1	1.2503 (4)	−0.31406 (18)	0.19870 (3)	0.0379 (3)	
F1	0.8185 (7)	−0.1764 (6)	0.24039 (7)	0.0389 (8)	
O1	0.5500 (7)	0.4407 (5)	0.12674 (7)	0.0216 (5)	
N1	0.9909 (6)	0.3622 (6)	0.13959 (7)	0.0183 (6)	
H1N1	1.1678	0.3812	0.1374	0.022*	
C1	0.6150 (9)	0.9292 (7)	0.09204 (10)	0.0206 (8)	
H1A	0.6939	0.9808	0.1110	0.025*	
C2	0.4185 (9)	1.0515 (6)	0.07644 (9)	0.0195 (8)	
H2A	0.3622	1.1843	0.0847	0.023*	
C3	0.3096 (8)	0.9750 (6)	0.04902 (9)	0.0158 (7)	
C4	0.3851 (9)	0.7798 (6)	0.03669 (10)	0.0185 (7)	
H4A	0.3046	0.7284	0.0179	0.022*	
C5	0.5801 (9)	0.6617 (6)	0.05240 (9)	0.0189 (7)	
H5A	0.6354	0.5291	0.0440	0.023*	
C6	0.6964 (9)	0.7335 (6)	0.08025 (10)	0.0182 (7)	
C7	0.9099 (8)	0.6042 (6)	0.09676 (10)	0.0204 (8)	
H7A	1.0466	0.7010	0.1061	0.025*	
H7B	1.0043	0.5142	0.0811	0.025*	
C8	0.7943 (8)	0.4641 (6)	0.12252 (9)	0.0157 (7)	
C9	0.9335 (9)	0.2242 (6)	0.16520 (9)	0.0179 (7)	
C10	0.7350 (10)	0.2685 (8)	0.18765 (11)	0.0268 (9)	
H10A	0.6262	0.3923	0.1858	0.032*	
C11	0.6968 (10)	0.1312 (10)	0.21275 (11)	0.0320 (10)	
H11A	0.5588	0.1590	0.2279	0.038*	
C12	0.8580 (11)	−0.0440 (8)	0.21570 (11)	0.0275 (9)	
C13	1.0540 (12)	−0.0902 (6)	0.19399 (9)	0.0228 (8)	
C14	1.0956 (9)	0.0427 (7)	0.16824 (9)	0.0211 (8)	
H14A	1.2313	0.0108	0.1530	0.025*	

### Atomic displacement parameters (Å^2^)

**Table:**

	*U*^11^	*U*^22^	*U*^33^	*U*^12^	*U*^13^	*U*^23^
Br1	0.01688 (15)	0.01907 (14)	0.02281 (16)	0.00264 (16)	0.00107 (16)	0.00387 (15)
Cl1	0.0564 (9)	0.0216 (5)	0.0357 (6)	0.0089 (5)	−0.0016 (6)	−0.0003 (4)
F1	0.0354 (18)	0.0496 (19)	0.0316 (14)	−0.0056 (16)	0.0028 (13)	0.0207 (14)
O1	0.0082 (11)	0.0314 (13)	0.0253 (13)	−0.0002 (14)	0.0008 (13)	0.0045 (11)
N1	0.0022 (14)	0.0290 (14)	0.0236 (14)	−0.0016 (13)	0.0004 (10)	0.0048 (14)
C1	0.020 (2)	0.0221 (16)	0.0193 (17)	−0.0013 (15)	0.0019 (15)	−0.0015 (14)
C2	0.017 (2)	0.0183 (15)	0.0229 (17)	0.0020 (16)	0.0024 (16)	−0.0017 (13)
C3	0.0116 (17)	0.0151 (14)	0.0206 (17)	−0.0001 (13)	0.0018 (14)	0.0030 (12)
C4	0.0139 (18)	0.0213 (16)	0.0202 (16)	0.0035 (14)	−0.0002 (14)	−0.0024 (13)
C5	0.0202 (19)	0.0135 (14)	0.0231 (16)	0.0030 (16)	−0.0007 (15)	−0.0006 (13)
C6	0.0142 (18)	0.0204 (16)	0.0200 (18)	0.0003 (15)	0.0013 (15)	0.0015 (14)
C7	0.0114 (19)	0.0263 (19)	0.0235 (17)	0.0004 (14)	0.0028 (14)	0.0065 (14)
C8	0.0067 (15)	0.0216 (16)	0.0187 (17)	−0.0004 (14)	−0.0027 (13)	−0.0020 (13)
C9	0.0082 (15)	0.0261 (16)	0.0194 (16)	−0.0035 (16)	−0.0021 (16)	0.0007 (13)
C10	0.018 (2)	0.038 (2)	0.025 (2)	0.0064 (19)	0.0039 (17)	0.0044 (17)
C11	0.019 (2)	0.051 (3)	0.026 (2)	0.009 (2)	0.0075 (17)	0.011 (2)
C12	0.027 (2)	0.033 (2)	0.023 (2)	−0.007 (2)	−0.0017 (18)	0.0093 (17)
C13	0.027 (2)	0.0179 (15)	0.0234 (17)	−0.0008 (18)	−0.0069 (19)	−0.0003 (12)
C14	0.018 (2)	0.0259 (18)	0.0190 (17)	0.0000 (16)	0.0040 (15)	−0.0017 (14)

### Geometric parameters (Å, º)

**Table:**

Br1—C3	1.910 (4)	C5—C6	1.391 (6)
Cl1—C13	1.723 (4)	C5—H5A	0.9500
F1—C12	1.356 (5)	C6—C7	1.503 (6)
O1—C8	1.222 (5)	C7—C8	1.518 (6)
N1—C8	1.369 (5)	C7—H7A	0.9900
N1—C9	1.423 (5)	C7—H7B	0.9900
N1—H1N1	0.8825	C9—C10	1.393 (6)
C1—C6	1.392 (6)	C9—C14	1.401 (6)
C1—C2	1.403 (6)	C10—C11	1.388 (7)
C1—H1A	0.9500	C10—H10A	0.9500
C2—C3	1.371 (6)	C11—C12	1.366 (7)
C2—H2A	0.9500	C11—H11A	0.9500
C3—C4	1.390 (5)	C12—C13	1.365 (7)
C4—C5	1.385 (6)	C13—C14	1.395 (6)
C4—H4A	0.9500	C14—H14A	0.9500
			
C8—N1—C9	123.6 (3)	C6—C7—H7B	109.0
C8—N1—H1N1	125.0	C8—C7—H7B	109.0
C9—N1—H1N1	111.2	H7A—C7—H7B	107.8
C6—C1—C2	121.1 (4)	O1—C8—N1	123.9 (4)
C6—C1—H1A	119.5	O1—C8—C7	122.9 (4)
C2—C1—H1A	119.5	N1—C8—C7	113.1 (3)
C3—C2—C1	118.5 (4)	C10—C9—C14	119.9 (4)
C3—C2—H2A	120.8	C10—C9—N1	122.7 (4)
C1—C2—H2A	120.8	C14—C9—N1	117.3 (4)
C2—C3—C4	122.0 (4)	C11—C10—C9	119.8 (4)
C2—C3—Br1	119.2 (3)	C11—C10—H10A	120.1
C4—C3—Br1	118.8 (3)	C9—C10—H10A	120.1
C5—C4—C3	118.7 (4)	C12—C11—C10	119.9 (4)
C5—C4—H4A	120.7	C12—C11—H11A	120.1
C3—C4—H4A	120.7	C10—C11—H11A	120.1
C4—C5—C6	121.3 (4)	F1—C12—C13	119.5 (4)
C4—C5—H5A	119.4	F1—C12—C11	119.2 (5)
C6—C5—H5A	119.4	C13—C12—C11	121.3 (4)
C5—C6—C1	118.6 (4)	C12—C13—C14	120.4 (4)
C5—C6—C7	120.5 (4)	C12—C13—Cl1	119.4 (3)
C1—C6—C7	120.9 (4)	C14—C13—Cl1	120.2 (4)
C6—C7—C8	113.1 (3)	C13—C14—C9	118.7 (4)
C6—C7—H7A	109.0	C13—C14—H14A	120.6
C8—C7—H7A	109.0	C9—C14—H14A	120.6
			
C6—C1—C2—C3	−0.7 (6)	C8—N1—C9—C10	−41.6 (6)
C1—C2—C3—C4	1.1 (6)	C8—N1—C9—C14	141.6 (4)
C1—C2—C3—Br1	−178.0 (3)	C14—C9—C10—C11	−0.7 (7)
C2—C3—C4—C5	−1.2 (6)	N1—C9—C10—C11	−177.4 (4)
Br1—C3—C4—C5	177.8 (3)	C9—C10—C11—C12	1.5 (8)
C3—C4—C5—C6	1.1 (6)	C10—C11—C12—F1	179.8 (5)
C4—C5—C6—C1	−0.7 (6)	C10—C11—C12—C13	−1.4 (8)
C4—C5—C6—C7	−179.3 (4)	F1—C12—C13—C14	179.3 (4)
C2—C1—C6—C5	0.5 (6)	C11—C12—C13—C14	0.5 (8)
C2—C1—C6—C7	179.1 (4)	F1—C12—C13—Cl1	−0.8 (6)
C5—C6—C7—C8	−95.2 (5)	C11—C12—C13—Cl1	−179.6 (4)
C1—C6—C7—C8	86.3 (5)	C12—C13—C14—C9	0.2 (7)
C9—N1—C8—O1	−2.9 (6)	Cl1—C13—C14—C9	−179.6 (3)
C9—N1—C8—C7	179.2 (4)	C10—C9—C14—C13	−0.1 (6)
C6—C7—C8—O1	8.2 (6)	N1—C9—C14—C13	176.7 (4)
C6—C7—C8—N1	−174.0 (3)		

### Hydrogen-bond geometry (Å, º)

**Table:**

*D*—H···*A*	*D*—H	H···*A*	*D*···*A*	*D*—H···*A*
N1—H1*N*1···O1^i^	0.88	1.97	2.844 (5)	172
C2—H2*A*···O1^ii^	0.95	2.58	3.321 (5)	135
C10—H10*A*···Cl1^iii^	0.95	2.67	3.583 (5)	160
C11—H11*A*···F1^iv^	0.95	2.52	3.443 (6)	165

Symmetry codes: (i) *x*+1, *y*, *z*; (ii) *x*, *y*+1, *z*; (iii) *x*−1, *y*+1, *z*; (iv) −*x*+1, *y*+1/2, −*z*+1/2.

## References

[bb1] Bruker (2009). *APEX2*, *SAINT* and *SADABS* Bruker AXS Inc., Madison, Wisconsin, USA.

[bb2] Cosier, J. & Glazer, A. M. (1986). *J. Appl. Cryst.* **19**, 105–107.

[bb3] Flack, H. D. (1983). *Acta Cryst.* A**39**, 876–881.

[bb4] Fun, H.-K., Quah, C. K., Narayana, B., Nayak, P. S. & Sarojini, B. K. (2011*a*). *Acta Cryst.* E**67**, o2926–o2927. 10.1107/S1600536811041110 PMC3247340 22219958

[bb5] Fun, H.-K., Quah, C. K., Narayana, B., Nayak, P. S. & Sarojini, B. K. (2011*b*). *Acta Cryst.* E**67**, o2941–o2942. 10.1107/S1600536811041468 PMC3247353 22219971

[bb6] Fun, H.-K., Quah, C. K., Nayak, P. S., Narayana, B. & Sarojini, B. K. (2012*a*). *Acta Cryst.* E**68**, o1385. 10.1107/S1600536812014869 PMC3344513 22590275

[bb7] Fun, H.-K., Quah, C. K., Nayak, P. S., Narayana, B. & Sarojini, B. K. (2012*b*). *Acta Cryst.* E**68**, o2461. 10.1107/S1600536812031595 PMC3414917 22904904

[bb8] Sheldrick, G. M. (2008). *Acta Cryst.* A**64**, 112–122. 10.1107/S0108767307043930 18156677

[bb9] Spek, A. L. (2009). *Acta Cryst.* D**65**, 148–155. 10.1107/S090744490804362X PMC2631630 19171970

